# Low DLCO Can Provide Insights into Treatment Response in PAH Patients Irrespective of the Reason for Its Decrease

**DOI:** 10.3390/life15101551

**Published:** 2025-10-03

**Authors:** Effrosyni Dima, Stylianos E. Orfanos, Vasileios Grigoropoulos, Dimitra Fasfali, Athina Mpatsouli, Natalia P. Zimpounoumi-Keratsa, Panagioula Niarchou, Athanasia Megarisiotou, Efstathia Prappa, Sotirios Xydonas, Anastasia Kotanidou, Ioanna Dimopoulou, Anastasia Anthi

**Affiliations:** 1 Pulmonary Hypertension Center of “Evangelismos” Hospital and 1st Department of Critical Care Medicine and Pulmonary Services, Medical School, National and Kapodistrian University of Athens, “Evangelismos” Hospital, 10676 Athens, Greece; vasilisgrig@hotmail.com (V.G.); dfasfali@gmail.com (D.F.); akotanid@med.uoa.gr (A.K.); idimop@med.uoa.gr (I.D.); anastasia.anthi1@gmail.com (A.A.); 2 Critical Care, Medical School, National and Kapodistrian University of Athens, 10676 Athens, Greece; sorfanos@med.uoa.gr; 3 Pulmonary Hypertension Center of “Evangelismos” Hospital, and Cardiology Department, “Evangelismos” Hospital, 10676 Athens, Greece; athmpatsouli@gmail.com (A.M.); nanapnzk@hotmail.com (N.P.Z.-K.); nargiouli@hotmail.com (P.N.); athamega@hotmail.com (A.M.); efiprappa@gmail.com (E.P.); sotxyd@gmail.com (S.X.)

**Keywords:** PAH, DLCO, phenotype, clinical characteristics, treatment

## Abstract

Group 1 of PAH patients encompasses patients with a diverse underlying etiological condition, having histological modifications that can affect gas exchange across the alveolar-capillary membrane, as reflected by decreased DLCO. Values of DLCO did not identify the exact reason for their decrease, but they can provide insights into the underlying pathobiology and prognosis of PAH patients. Our aim was to explore whether PAH patients with low DLCO constitute a different subpopulation and describe their characteristics and response to treatment. A total of 69 PAH patients were studied retrospectively and divided into two groups: *group 1*: DLCO ≥ 45% and *group 2*: DLCO < 45%. IPAH and PAH-CTD mainly constituted our population. The proportion of IPAH to PAH-CTD was almost the same between the two groups. Patients in *group 2* were older (66.83 ± 11.61 vs. 59.27 ± 111.90, *p* = 0.035), mostly male (47.5% vs. 11.5% *p* = 0.008), and ever smokers (59% vs. 22%, *p* = 0.049). They mainly had WHO-FC III (68% vs. 32%) and had received more advanced therapy (40% on triple combination therapy vs. 16%). The two groups had similar mean PAP (*group 1* = 32 (22.00–38.00) vs. *group 2* = 35 (28.50–48.50) mmHg), while PVR was higher in *group 2* (6.49 (4.10–9.52) vs. 3.61 (2.95–5.22) WU). In *group 2*, neither IPAH nor PAH-CTD patients improved WHO-FC, 6MWD, or NT-proBNP after treatment. In our center, PAH patients with low DLCO had some distinct clinical characteristics, such as poor prognosis and poor treatment response to vasodilatory therapy. Understanding the role of DLCO in both phenotyping PAH patients and in treatment response would be useful in guiding therapeutic approaches, especially now that new therapeutic targets are involved in PAH treatment.

## 1. Introduction

Pulmonary Arterial Hypertension (PAH) is a progressive disease characterized by increased pulmonary vascular resistance, leading to right heart failure and significant morbidity. The Group of PAH patients encompasses patients with a diverse underlying etiological condition, having distinguished histological modifications [1,2] such as augmented contractility of pulmonary arterioles, endothelial dysfunction, disrupted signaling pathways and changes in endothelial and smooth muscle cells [3]. Despite their different etiologies, guidelines suggest a similar therapeutic approach, based on data showing that these patients can have a beneficial treatment effect regarding the reduction in the risk of morbidity/mortality or time to clinical worsening [4,5,6]. Idiopathic PAH (IPAH) and connective tissue disorders (CTDs) are common etiologies of PAH patients [7,8].

The Diffusing Capacity of the Lungs for Carbon Monoxide (DLCO) reflects the efficiency of gas transfer from the alveoli to the pulmonary circulation and depends on both the integrity of the alveolar-capillary membrane and the pulmonary microvasculature. A reduction in DLCO can therefore occur in a variety of conditions, including interstitial lung disease, emphysema, and pulmonary vascular disorders [9]. In PAH, DLCO appears to reflect the degree of pulmonary vascular bed alterations [10,11,12] where the main pathological changes occur. Furthermore, DLCO can provide insights into the underlying pathobiology [13,14,15,16,17], as well as the prognosis of PAH patients regardless of the PAH etiology [18,19,20,21,22].

Recently, a registry analysis in patients with IPAH indicates that patients with reduced DLCO (<45%) constitute a different phenotype, the lung phenotype of IPAH patients. These patients have worse survival outcomes despite being treated with PAH specific drugs [23]. Data in the literature regarding the response to current PAH therapies addressing the three classic pathways (PDE5i, ERA and prostanoids) are limited, and findings suggest that these patients can still experience hemodynamic and cardiac function improvements following treatment [24].

Our purpose was to explore whether PAH patients, in general, and not just those with IPAH and low DLCO in our center, constitute a different subpopulation and describe their clinical and hemodynamic characteristics, as well as their response to treatment. In other words, our purpose was to explore whether low DLCO, irrespective of the reason for its decrease in PAH patients, can recognize patients with specific characteristics and responses to treatment. Understanding the role of DLCO in PAH phenotyping could guide personalized therapeutic approaches.

## 2. Methods

We retrospectively studied PAH patients seen at the Evangelismos Hospital PH center (Athens, Greece) between September 2022 and November 2024.

PAH diagnosis was made by our center physicians and included a multidisciplinary team assessment.

Diagnosis was based on right heart catheterization. PAH was diagnosed in patients with a mean pulmonary arterial pressure (PAP) > 20 mmHg, pulmonary arterial wedge pressure (PAWP) ≤ 15 mmHg and pulmonary vascular resistance > 2 Wood Units (WU), not explained by an underlying parenchymal lung disease, chronic obstructive pulmonary disease (COPD), chronic thromboembolic pulmonary hypertension (CTEPH), or multiple unclear mechanisms [1,25].

The patients were also evaluated clinically and by echocardiography; additionally, among others, chest computed tomography (CT) (HRCT and/or CTPA), perfusion lung scanning and pulmonary function testing were performed in order to obtain the right diagnosis; they were classified as PAH patients—Group 1—according to the most recent ESC/ERS guidelines [1]. Although chest CT was performed, we did not systematically re-review scans for this study.

Patients diagnosed with pulmonary hypertension due to left heart disease, pulmonary disease, chronic thromboembolic disease and multiple and unclear mechanisms were excluded. Furthermore, patients categorized as having PAH due to congenital heart diseases were also excluded. Only adult patients (>18 years old) were included.

At the time of diagnosis, functional capacity was assessed by the World Health Organization functional class (WHO-FC) and 6 min Walking Distance (6MWD); N-terminal prohormone of Brain Natriuretic Peptide (NT-proBNP) was also measured.

Pulmonary function (FEV1, FVC and FEV1/FVC) [26] and single-breath carbon monoxide diffusing capacity [9] were measured (Jaeger MasterScreen™ PFT, CareFusion, Hoechberg, Germany) according to international standards.

Patients, after the time of diagnosis, received PAH-specific therapy prescribed by our center physicians according to contemporary guidelines, consisting of phosphodiesterase type-5 inhibitors (PDE5is), endothelin receptor antagonists (ERAs), soluble guanylate cyclase (sGC) stimulator and prostanoids. Most of our patients received a combination treatment. The first clinical evaluation was three months after therapy initiation in stable patients or when needed in unstable patients. The reported reevaluation values in our manuscript were recorded after three months of stable/unchanged therapy.

Patients’ response to treatment was assessed by WHO-FC, 6MWD and NT-proBNP values, as suggested by the current ESC/ERS guidelines pertaining to follow-up assessment.

The study was approved by the Evangelismos Hospital Research Ethics Committee (4783/22-2-2022) and conducted in compliance with the Helsinki Declaration. Informed written consent was obtained from all patients included in the study.

### Statistical Analysis

Data were presented as mean ± standard deviation for continuous variables with normal distribution, and as median and interquartile ranges for non-normally distributed variables. Categorical variables were presented as frequencies and percentages (%). Continuous variables were compared using the *t*-test for independent samples or the Mann–Whitney U test, while the chi-square test or the Fisher exact test was used to assess categorical variables. Statistical tests were pairwise when appropriate, using the McNemar test for categorical variables and the paired *t*-test for continuous variables.

A *p*-value < 0.05 was considered statistically significant in this study. Data were analyzed using SPSS version 25.0 (IBM SPSS Statistics for Windows, Version 25.0.).

## 3. Results

A total of 69 PAH patients have been followed up with and treated in our center over 26 months. 42% (n = 29) have been diagnosed as IPAH, 55% (n = 38) as having PAH due to CTD (mostly systemic sclerosis (76%)) and 3% (n = 2) due to portal hypertension and HIV. In our study population, no patient has been categorized as suffering from PAH due to pulmonary veno-occlusive disease (PVOD); patients with congenital heart disease were excluded due to the known effect of this clinical condition on DLCO and their different clinical course.

PAH patients were divided into two groups according to their DLCO predicted value (*group 1* = DLCO ≥ 45% and *group 2* = DLCO < 45%). The cut-off value of 45% predicted for DLCO was based on previous studies in order for our results to be comparable [23,27]. DLCO measurement was performed during the patient’s initial evaluation and before treatment initiation, using the single-breath method. We have used the predicted DLCO values corrected to hemoglobin. The main clinical characteristics are seen in Table 1, revealing the following results.

IPAH and PAH due to CTD constitute the majority of PAH patients in our cohort. The proportion of IPAH to PAH-CTD is almost the same in our two group. Patients with PAH due to portal hypertension or HIV are all in *group 1*. Patients in *group 2* were older (66.83 ± 11.61 vs. 59.27 ± 111.90, *p* = 0.035), mostly male (47.5% vs. 11.5% *p* = 0.008) and ever smokers (59% vs. 22%, *p* = 0.049). *Group 2* suffered more often from comorbidities, such as arterial hypertension, diabetes mellitus and coronary artery disease; however, the difference between the two groups did not reach statistical significance. *Group 1* patients mainly had WHO-FC II (68%), whereas *group 2* had FC III (61%). A total of 63% of *group 1* and 52% of *group 2* patients received oral combination therapy (PDE5i or sGC stimulator + ERA), whereas 40% of *group 2* received triple combination therapy, including a parental prostanoid (PDE5i + ERA + prostanoid), versus 16% of *group 1*. Therapy was escalated early, and the majority of patients were on stable therapy after six to nine months of the initial diagnosis. In total, 20% of *group 1* and 8% of *group 2* received monotherapy with PDE5i or ERA. Among those on monotherapy, 75% of *group 1* received ERA, whereas all of *group 2* received PDE5i.

PFTs are presented as the % of a predicted value in order to allow a comparison with previous studies. Spirometric values (FEV_1_, FVC) did not differ significantly between our *groups*.

The main hemodynamic and echocardiographic differences in the two groups are seen in Table 2. There was no statistically significant difference in mean pulmonary artery pressure (mean PAP) between the two groups (*group 1* = 32 (22.00–38.00) mmHg vs. *group 2* = 35 (28.50–48.50) mmHg, *p* = 0.063), similarly in pulmonary arterial wedge pressure (PAWP) (*group 1*= 11.00 ± 0.98 mmHg vs. *group 2* = 9.90 ± 2.67 mmHg, *p* = 0.317) and cardiac index (CI) (*group 1* = 2.58 ± 0.79 L/min/m^2^ vs. *group 2* = 2.30 ± 0.58 L/min/m^2^, *p* = 0.077). Statistically significant differences were found in pulmonary vascular resistance (PVR) (*group 1* = 3.61 (2.95–5.22) WU vs. *group 2* = 6.49 (4.10–9.52) WU, *p* = 0.006) and stroke volume index (SVI) (*group 1* = 40.28 ± 13.24 mL/m^2^ vs. *group 2* = 30.88 ± 7.33 mL/m^2^, *p* = 0.028).

Echocardiographic parameters that were statistically significantly different were tricuspid annular plane systolic excursion (TAPSE) (*group 1* = 22 (20.5–24) mm vs. *group 2* = 18 (16.5–20) mm, *p* = 0.003), tricuspid annular plane systolic excursion/right ventricular systolic pressure (TAPSE/RVSP) (*group 1* = 0.48 ± 0.17 mm/mmHg vs. *group 2* = 0.32 ± 0.14 mm/mmHg, *p* = 0.004), tricuspid valve maximal velocity (TRVmax) (*group 1* = 3.23 ± 0.64 m/sec vs. *group 2* = 3.64 ± 0.55 m/s, *p* = 0.039) and pulmonary artery diameter (Pad) (*group 1* = 24.76 ± 5.37 mm vs. *group 2* = 28.22 ± 3.37 mm, *p* = 0.032).

Our patients have been treated according to their physicians, as seen in Table 1. In the follow-up WHO-FC, 6MWT and NT-proBNP were reassessed. The results are seen in Table 3, Table 4 and Table 5 and are pairwise. *Group 1* patients had improved WHO-FC, 6MWT and NT-proBNP, whereas *group 2* did not show significant improvement. In *group 2,* neither IPAH nor PAH-CTD patients improved their clinical status regarding 6MWD and NT-proBNP.

## 4. Discussion

In our center, PAH patients with DLCO < 45% tend to be older, male and smokers or ex-smokers. They have worse functional status, parameters and indices that carry a worse prognosis and respond worse to treatment. They also have spiromertric indices indicating well preserved pulmonary function that does not differ in comparison with patients with DLCO ≥ 45%. We did not evaluate chest CT scans for underlying emphysema and fibrosis based on the above-mentioned normal spirometric values. Having known that spirometry does not correlate to HRCT findings, we cannot exclude that our patients, especially those with a smoking history and connective tissue disease, may also have some degree of parenchymal lung disease that would not exclude them from the PAH Group. Furthermore, the median PVR value of *group 2* (>5 WU) suggests a certain pulmonary arterial component.

In our study, the two patient groups have the same hemodynamic impairment in terms of mean PAP. Mean PAP in *group 2*, although it has been higher, did not reach a statistically significant difference when compared to *group 1*. However, PVR was statistically different between the two groups, implying a more severe disease in *group 2*. A larger sample of patients may be needed to clarify it.

Based on the marginal hemodynamic differences between our two groups, although not statistically significant, we consider that PH severity could be an obvious explanation for our results. Nevertheless, some interesting points emerged from our findings, as described below:

In our study, all PAH patients with low DLCO have clinical characteristics similar to previously studied IPAH patients with low DLCO, meaning that they tend to be older, male and with a smoking history. First, Trip et al. studied IPAH patients with severe pre-capillary PH and DLCO < 45%. Most of these patients were men and had a smoking history, but spirometric indices were relatively well-preserved, and 32% had normal findings on CT [27]. Similarly, analysis from COMPERA and ASPIRE patients showed that IPAH patients with a ‘lung phenotype’ had normal or near normal spirometry, and a severe reduction in DLCO; they were older and had a lower proportion of female patients than expected in IPAH, while they were also all ever smokers [23]. In our population, approximately 60% of patients have PAH-CTD, meaning that both IPAH and PAH-CTD patients with reduced DLCO < 45% share similar clinical characteristics.

Data in the literature have shown an involvement of angiotensin converting enzyme 1 (ACE 1) in PAH [28,29,30,31]; a loss of functional capillary surface area in vivo in both IPAH and PAH-CTD patients have been detected, with a correlation with DLCO levels only in PAH-CTD patients and a more severe pulmonary capillary endothelial metabolic dysfunction in patients with PAH-CTD compared to those with IPAH [32]. Our data may indicate that, despite the different pathogenesis of endothelial function between the two types of PAH, some common factors may further influence the endothelial dysfunction leading to PAH patients with severely decreased DLCO.

Furthermore, Trip et al. found in their study that on multivariate regression analysis, age and the number of smoking pack-years were independently associated with a severely reduced DLCO [27]. Smoking-related vasculopathy is a relatively recent theory [33], where smoking is thought to be a cause of reduced DLCO in PAH patients, even in the absence of any obvious emphysema or interstitial lung disease upon chest imaging [34]. In murine experimental models, animals which were exposed to tobacco smoke had pulmonary vascular damage with a loss of capillaries that preceded the development of emphysema [34]. Although male sex has not been correlated to decreased DLCO, male gender is correlated to worse survival, even more to worse right ventricle (RV) adaptability as compared to female PAH patients [35,36,37].

Another finding of our study is that *group 2* has a worse response to PAH therapy, although these patients receive more frequently triple combination PAH-specific therapy, including parental prostanoids; this underlies, in part, their poor prognosis. In our study, *group 2* patients have parameters that carry a worse prognosis, namely WHO-FC, 6MWT, NT-proBNP, TAPSE/RSVP and SVI. Once again, PH severity may be an explanation, and one could hypothesize that the poor response to treatment of our *group 2* is in part due to the known worse prognosis of PAH-CTD compared to IPAH [21,38,39]. However the percentage of PAH-CTD is similar between the two groups. Until now, studies indicating poor response to treatment in PAH-CTD did not have any information regarding DLCO [40,41]. Data on PAH-specific therapy response regarding DLCO refer to the comparison of PAH-CTD patients and CTD patients with PH due to lung disease and are scarce [42]. Of note in our population, the same poor response was detected in IPAH patients of *group 2*, who did not have more severe hemodynamics compared to IPAH patients of *group 1* (not seen in the results). Previous studies have demonstrated the same response in IPAH patients with low DLCO and/or mild lung parenchyma lung disease, resembling PH patients with lung disease (Group 3) [23,43]. Our results need further investigation and confirmation from other studies.

The presence of even mild parenchymal lung disease affects both IPAH and PAH-CTD patients’ outcome [42,43]. The possibility that mild lung parenchyma abnormalities are the reason for the poor response of our *group 2* patients cannot be answered by our study. However, our purpose was to explore the meaning of low DLCO in PAH patients in terms of clinical characteristics, hemodynamic severity, and response to treatment, irrespective of the reason for the DLCO decrease. Can chest CT aid in the further understanding of phenotyping and treating of such PAH patients? This needs more investigation.

Our study is a retrospective, single-center study and has some limitations. The small number of patients in each group has the risk of not recognizing differences and correlations between groups, due to underlying beta error. The small number and missing values did not allow us to proceed to further statistical analysis to determine the characteristics that were independently associated with a severely reduced DLCO and poor response to therapy. We reported PFTs as %predicted values. Since a recent ATS/ERS official statement recommend z scores for their interpretation, future studies should adopt them [26]. Furthermore, we did not evaluate CT scans because, among others, they were performed in various centers, leading to a heterogeneity in their quality. As a result, some mild structural abnormalities may have gone undetected, and this limitation may partly explain the associations observed with low DLCO. Finally, we did not analyze hemodynamic or echocardiographic post-treatment data, and we preferred the analysis to be based on more clinically meaningful parameters and easy, widely available tests.

Taken together, our results indicate that PAH patients with low DLCO, irrespective of the reason for the DLCO decrease, constitute a subgroup that was mainly older, male and smokers; this subgroup was also associated with worse functional class and a poorer response to vasodilatory therapy. As our study is retrospective and observational, these findings should be interpreted as associations rather than evidence of causation. However, they shed light on the poor response to vasodilatory therapy of PAH patients with low DLCO, regardless of the underlying cause of the reduction.

Although risk stratification, functional class as well as PH severity constitute major parameters that determine the therapeutic decisions, the worse response to vasodilatory treatment of patients with low DLCO may generate the question of an alternative therapeutic approach in this patient group. Certainly, further studies are needed to determine if DLCO, besides being a prognostic factor for PAH, can also phenotype PAH patients according to their response to vasodilatory treatment. It would be of interest to further explore whether PAH patients with low DLCO, mainly patients with IPAH and CTD, have a worse response to therapy, especially now that new therapeutic targets are involved in the treatment of PAH patients.

## Figures and Tables

**Table 1:** Patients’ main characteristics and received treatment.

	*Group 1* (n = 33)	*Group 2* (n = 36)	*p*
**IPAH%/PAH-CTD%**	32/63	39/61	0.560
**Age years**	59.27 ± 11.90	66.83 ± 11.61	0.035
**Male %**	12	47	0.008
**BMI kg/m^2^**	28.32 (26.14–34.01)	25.81 (23.55–30.68)	0.128
**WHO-FC**			0.016
WHO-FCII %	68	30	
WHO-FCIII %	32	61	
WHO-FCIV %	0	9	
**Ever smokers %**	22	59	0.049
**Comorbidities %**	32	43.5	0.632
**PFTs**			
FEV_1_ %pred	90 (74.2–105.8)	81.6 (59.3–92.0)	0.073
FVC %pred	99 (79.3–103.6)	92 (60.6–100.0)	0.189
FEV_1_/FVC %	79.8 (75–84)	74 (65–82.6)	0.044
TLC %pred	83.9 (76.8–91.4)	68.8 (56.0–78.0)	0.002
**6MWD m**	417.88 ± 77.61	285.45 ± 125.54	0.001
**NT-proBNP pg/mL**	939.00 ± 1565.54	1126.22 ± 1491.42	0.452
**Treatment**			0.208
Monotherapy (PDE5i or ERA)	21%	8%	
Double oral combination (PDE5i/sGC stimulator + ERA)	63%	52%	
Triple combination therapy (PDE5i/sGC stimulator + ERA + prostanoid)	16%	40%	

*Group 1*: PAH patients with DLCO ≥ 45%, *group 2*: PAH patients with DLCO < 45%, IPAH: Ιdiopathic Pulmonary Arterial Hypertension, PAH-CTD: Pulmonary Arterial Hypertension associated with Connective Tissue Disorders, WHO-FC: World Health Organization Functional Class, PFTs: Pulmonary Function Tests, FEV_1_: Forced Expiratory Volume in 1 s, FVC: Forced Vital Capacity, TLC: Total Lung Capacity, 6MWD: 6 min Walking Distance, NT-proBNP: N-terminal prohormone of Brain Natriuretic Peptide, PDE5i: Phosphodiesterase type-5 Inhibitor, ERA: Endothelin Receptor Antagonist, sGC: soluble Guanylate Cyclase.

**Table 2:** Hemodynamic and echocardiographic characteristics.

	*Group 1*	*Group 2*	*p*
***RHC***			
RAP mmHg	7.05 ± 0.75	6.18 ± 0.74	0.405
Mean PAP mmHg	32 (22.00–38.00)	35 (28.50–48.50)	0.063
PAWP mmHg	11.00 ± 0.98	9.90 ± 2.67	0.317
PVR WU	3.61 (2.95–5.22)	6.49 (4.10–9.52)	0.006
CI L/min/m^2^	2.58 ± 0.79	2.30 ± 0.58	0.077
SVI mL/m^2^	40.28 ± 13.24	30.88 ± 7.33	0.028
**Echocardiography**			
TAPSE mm	22 (20.5–24)	18 (16.5–20)	0.003
RVSP mmHg	50.26 ± 20.96	62.55 ± 18.10	0.059
TAPSE/RVSP mm/mmHg	0.48 ± 0.17	0.32 ± 0.14	0.004
TRVmax m/s	3.23 ± 0.64	3.64 ± 0.55	0.039
RA cm^2^	19.18 ±6.81	19.35 ± 5.27	0.939
Pad mm	24.76 ± 5.37	28.22 ± 3.37	0.032

*Group 1*: PAH patients with DLCO ≥ 45%, *group 2*: PAH patients with DLCO < 45%, RHC: Right Heart Catheterization, RAP: Right Atrium Pressure, PAP: Pulmonary Artery Pressure, PAWP: Pulmonary Arterial Wedge Pressure, PVR: Pulmonary Vascular Resistance, CI: Cardiac Index, SVI: Stroke Volume Index, TAPSE: Tricuspid Annular Plane Systolic Excursion, RVSP: Right Ventricular Systolic Pressure, TRVmax: Tricuspid Valve maximal Velocity, Pad: Pulmonary artery diameter.

**Table 3:** WHO-FC assessment before and after treatment in the two groups.

WHO-FC	Before Treatment	After Treatment	*p*
***group 1***			0.023
WHO-FCII	68%	76%	
WHO-FCIII	32%	24%	
WHO-FCIV	0%	0%	
***group 2***			0.19
WHO-FCII	30%	30%	
WHO-FCIII	61%	62%	
WHO-FCIV	9%	8%	

*Group 1*: PAH patients with DLCO ≥ 45%, *group 2*: PAH patients with DLCO < 45%, WHO-FC: World Health Organization Functional Class.

**Table 4:** 6MWD before and after treatment in the two groups.

6MWD m	Before Treatment	After Treatment	*p*
***group 1***	417.88 ± 77.61	457.77 ± 56.17	0.023
***group 2***	285.45 ± 125.54	294.63 ± 132.18	0.834
			
*group 2 IPAH*	215.75 ± 167.24	198.75 ± 123.31	0.899
*group 2 PAH-CTD*	325.28 ± 84.6	346.57 ± 70.10	0.201

*Group 1*: PAH patients with DLCO ≥ 45%, *group 2*: PAH patients with DLCO < 45%, 6MWD: 6 min Walking Distance, IPAH: Ιdiopathic Pulmonary Arterial Hypertension. PAH-CTD: Pulmonary Arterial Hypertension associated with Connective Tissue Disorders.

**Table 5:** NT-proBNP before and after treatment in two groups.

NT-proBNP pg/mL	Before Treatment	After Treatment	*p*
***group 1***	558.70. ± 647.73	358.48 ± 204.83	0.045
***group 2***	1126.22 ± 1491.42	1476.13 ± 2117.22	0.210
			
*group 2 IPAH*	1232 ± 1077.96	1465 ± 949.51	0.155
*group 2 PAH-CTD*	1051.79 ± 1778.97	993.83 ± 1770.33	0.678

*Group 1*: PAH patients with DLCO ≥ 45%, *group 2*: PAH patients with DLCO < 45%, NT-proBNP: N-terminal prohormone of Brain Natriuretic Peptide, IPAH: Ιdiopathic Pulmonary Arterial Hypertension. PAH-CTD: Pulmonary Arterial Hypertension associated with Connective Tissue Disorders.

## Data Availability

Data are available upon reasonable request.

## Abbreviations

PAH: Pulmonary Arterial Hypertension, IPAH: Ιdiopathic Pulmonary Arterial Hypertension, PAH-CTD: Pulmonary Arterial Hypertension associated with Connective Tissue Disorders, DLCO: Diffusing Capacity of the Lungs for Carbon Monoxide, WHO-FC: WHO Functional Class, PAP: Pulmonary Artery Pressure, PVR: Pulmonary Vascular Resistance, 6MWD: 6 min Walking Distance, NT-proBNP: N-terminal Prohormone of Brain Natriuretic Peptide.
